# Proteomic and Biochemical Comparison of the Cellular Interaction Partners of Human VPS33A and VPS33B

**DOI:** 10.1016/j.jmb.2018.05.019

**Published:** 2018-07-06

**Authors:** Morag R. Hunter, Geoffrey G. Hesketh, Tomasz H. Benedyk, Anne-Claude Gingras, Stephen C. Graham

**Affiliations:** 1 Department of Pathology, University of Cambridge, Tennis Court Road, Cambridge CB2 1QP, UK; 2 Lunenfeld-Tanenbaum Research Institute, Mount Sinai Hospital, Toronto M5G 1X5, Canada; 3 Department of Molecular Genetics, University of Toronto, Toronto, Canada

**Keywords:** BFDR, Bayesian false discovery rate, CHEVI, class C homologs in endosome–vesicle interaction, CORVET, class C core vacuole/endosome tethering, EARP, endosome-associated recycling protein, GARP, Golgi-associated retrograde protein, HEK293T, human embryonic kidney 293T, HOPS, homotypic fusion and vacuole protein sorting, PI3KC3, class III phosphatidylinositol 3-kinase, XLID, X-linked intellectual disability, Sec1/Munc18, SPE-39, VIPAS39, VPS16B, X-linked intellectual disability (XLID)

## Abstract

Multi-subunit tethering complexes control membrane fusion events in eukaryotic cells. Class C core vacuole/endosome tethering (CORVET) and homotypic fusion and vacuole protein sorting (HOPS) are two such complexes, both containing the Sec1/Munc18 protein subunit VPS33A. Metazoans additionally possess VPS33B, which has considerable sequence similarity to VPS33A but does not integrate into CORVET or HOPS complexes and instead stably interacts with VIPAR. It has been recently suggested that VPS33B and VIPAR comprise two subunits of a novel multi-subunit tethering complex (named “CHEVI”), perhaps analogous in configuration to CORVET and HOPS. We utilized the BioID proximity biotinylation assay to compare and contrast the interactomes of VPS33A and VPS33B. Overall, few proteins were identified as associating with both VPS33A and VPS33B, suggesting that these proteins have distinct sub-cellular localizations. Consistent with previous reports, we observed that VPS33A was co-localized with many components of class III phosphatidylinositol 3-kinase (PI3KC3) complexes: PIK3C3, PIK3R4, NRBF2, UVRAG and RUBICON. Although VPS33A clearly co-localized with several subunits of CORVET and HOPS in this assay, no proteins with the canonical CORVET/HOPS domain architecture were found to co-localize with VPS33B. Instead, we identified that VPS33B interacts directly with CCDC22, a member of the CCC complex. CCDC22 does not co-fractionate with VPS33B and VIPAR in gel filtration of human cell lysates, suggesting that CCDC22 interacts transiently with VPS33B/VIPAR rather than forming a stable complex with these proteins in cells. We also observed that the protein complex containing VPS33B and VIPAR is considerably smaller than CORVET/HOPS, suggesting that the CHEVI complex comprises just VPS33B and VIPAR.

## Highlights

• VPS33A and VPS33B co-localize with distinct sets of cellular proteins.
• VPS33A co-localizes with PI3KC3 complex members.
• VPS33B interacts directly with CCDC22, a member of the CCC complex.
• VPS33B and VIPAR do not assemble into a larger stable multi-subunit tethering complex.

## Graphical abstract

**Figure:**
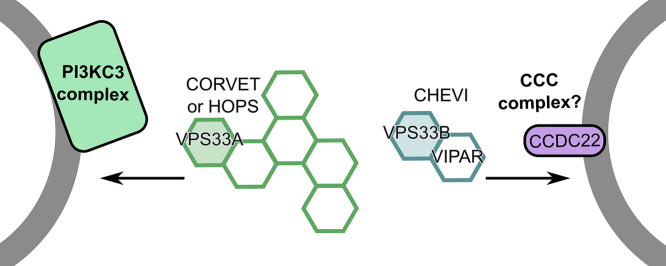

## Introduction

Membrane trafficking in eukaryotic cells is tightly controlled by a range of proteins, including markers of membrane identity (such as small GTPases) and multi-subunit tethering complexes. Tethering complexes, containing Sec1/Munc18 protein subunits, are soluble cytoplasmic proteins that work together with SNAREs to fuse membranes [1]. One such Sec1/Munc18 protein is Vps33, found in all eukaryotes as a subunit of class C core vacuole/endosome tethering (CORVET) and homotypic fusion and vacuole protein sorting (HOPS) complexes [2]. The mammalian HOPS and CORVET complexes share four common components, VPS33A, VPS16, VPS18 and VPS11, and have two distinct components, TRAP-1 and VPS8 (CORVET), or VPS39 and VPS41 (HOPS) [3], [4], [5]. CORVET is active on Rab5-positive membranes (early endosomes) [3], [6] and HOPS on Rab7-positive membranes (late endosomes, autophagosomes, lysosomes) [7], [8], [9].

In metazoans, there are two Vps33 homologs [10], with the functions of yeast Vps33 being carried out by VPS33A. Metazoan VPS33B shares 30% amino acid sequence identity with VPS33A, but cannot integrate into CORVET or HOPS complexes [3], [4], [8], [11], [12]. Instead, VPS33B has been observed on Rab10- and Rab25-positive membranes, functioning in post-Golgi trafficking [13], and on Rab11A-positive membranes acting in an apical recycling pathway [14].

Mutations in human VPS33A and VPS33B produce distinct phenotypes. Recently, a small population of patients have been identified with a single mutation in VPS33A (R498W) and a mucopolysaccharidosis-like phenotype [15], [16]. Although the mechanism is as yet unclear, this mutation results in over-acidification of lysosomes and an inability to catabolize glycosaminoglycans. This phenotype is quite different from that of patients with VPS33B mutations. VPS33B forms a complex with VIPAR (also known as SPE-39, VIPAS39 or VPS16B), and mutations in either of these proteins can cause arthrogryposis, renal dysfunction, and cholestasis (ARC) syndrome [14]. Arthrogryposis, renal dysfunction, and cholestasis syndrome is a multi-system disorder, with some symptoms attributable to the known functions of VPS33B and VIPAR in apical recycling pathways [14], post-Golgi collagen processing [13], and α-granule formation in megakaryocytes [17], [18]. Furthermore, mutations in VPS33B that affect its interactions with Rab proteins cause autosomal recessive keratoderma–ichthyosis–deafness syndrome [19]. The differences in phenotype conferred by mutations in VPS33A and VPS33B confirm that these proteins act in distinct cellular pathways.

A recent review suggested that VPS33B and VIPAR were members of a multi-subunit membrane tethering complex with an analogous organisation to CORVET and HOPS [20]. This hypothetical complex was called “class C homologs in endosome–vesicle interaction” (CHEVI) and it was postulated that CHEVI may comprise a dimer of the two currently known subunits (VPS33B and VIPAR) or that additional subunits may bind VPS33B/VIPAR to form a larger multi-subunit tethering complex, akin to HOPS/CORVET [20]. Here, we have used a quantitative proteomic approach in an attempt to identify members of the proposed CHEVI complex and to further investigate the differences between the cellular membranes with which VPS33A and VPS33B associate as part of their cognate multi-subunit tethering complexes. We identified known interactors of VPS33A and VPS33B, and novel VPS33B interactors, but did not find evidence for the existence of other members of a stable CHEVI complex beyond VPS33B and VIPAR.

## Results

**Fig. 1:**
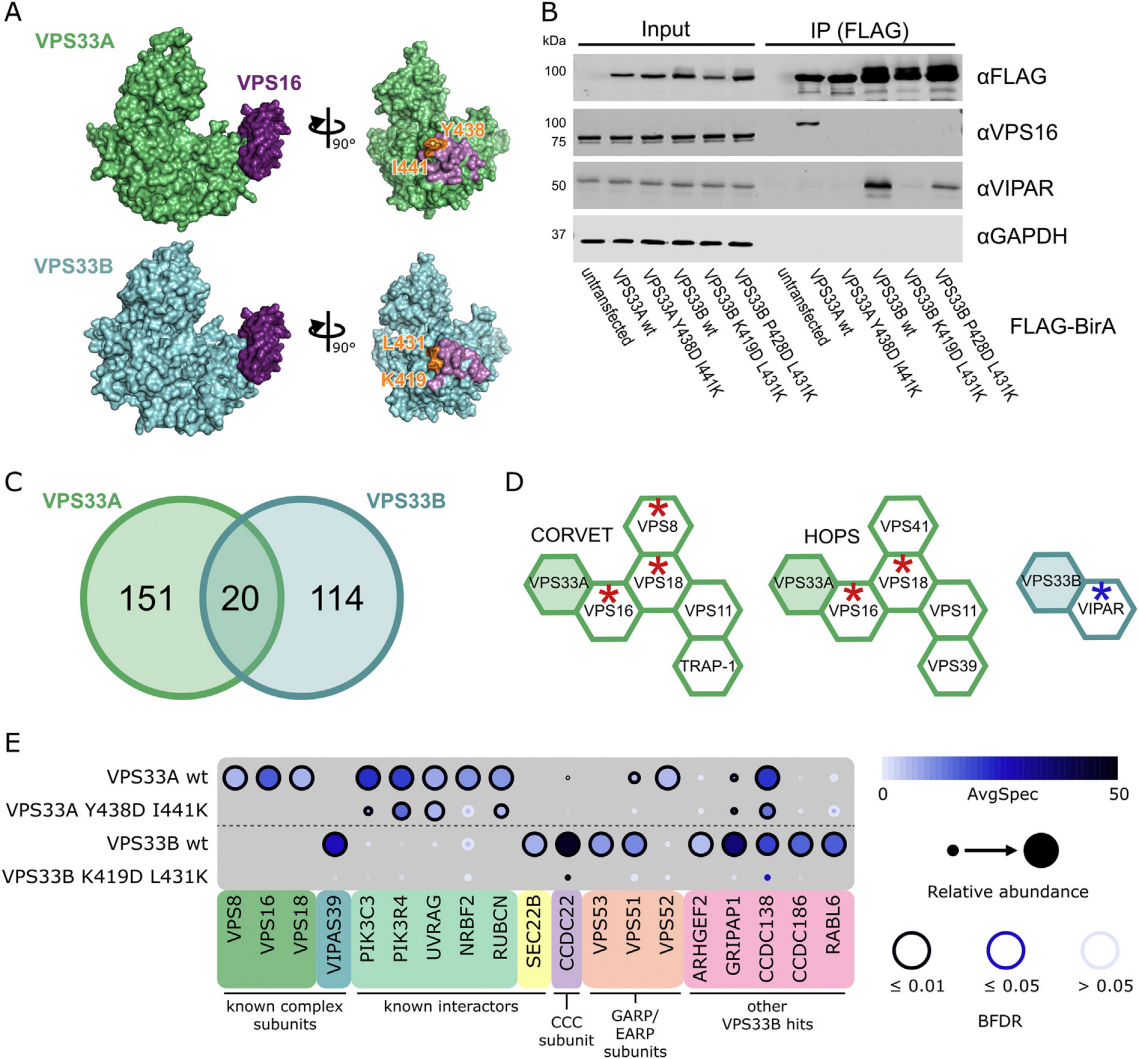
BioID results for VPS33A and VPS33B. (A) Crystal structure of VPS33A in complex with VPS16 residues 642–736 (top) and a homology model of VPS33B docked onto this complex (bottom). Residues shown in orange are mutated in panel B. Molecular graphics were generated using PyMOL (Schrodinger LLC). (B) HEK293 Flp-In T-REx cells were stably transfected with VPS33A and VPS33B (wild-type and mutant) constructs with C-terminal FLAG-BirA* tags and expression was induced with tetracycline. After immunoprecipitation (IP), samples were immunoblotted for the indicated endogenous protein. (C) Distribution of proteins identified by BioID with a BFDR of ≤ 0.01 and at least twofold enrichment in the wild-type sample over the negative-control mutant sample. (D) CORVET, HOPS and VPS33B–VIPAR (CHEVI) complexes, with subunits identified in BioID results marked with red asterisks (VPS33A as bait) or blue asterisk (VPS33B as bait). (E) Selected BioID results, shown as dot plots [59]. The spectral counts for each indicated prey protein are shown as AvgSpec. VIPAS39 = VIPAR, RUBCN = RUBICON. A complete list of the proximal proteins for each bait is available in the supplementary data file.

It is known that VPS33A must interact with VPS16 in order to be recruited to HOPS and to localize correctly to target membranes [8], [11], [21]. Mutation of two residues of VPS33A (Y438D and I441K) is sufficient to prevent the interaction between VPS33A and VPS16, and therefore inhibit HOPS function [8]. We utilized this construct as a negative control for VPS33A localization in our proteomics assays. The VPS33B–VIPAR complex is likely to closely resemble the VPS33A/VPS16 interaction [13]. We therefore docked a model of VPS33B generated using I-TASSER [22] onto the structure of human VPS33A in complex with VPS16 [11] to identify point mutations that may disrupt the VPS33B–VIPAR interaction, thus preventing correct VPS33B localization within the cell (Fig. 1A).

In order to compare VPS33A and VPS33B, we utilized the proximity-dependent biotinylation (BioID) assay [23]. This assay utilizes an abortive biotin ligase, BirA*, which releases an active intermediate of biotin (biotinoyl-5′-AMP) that can covalently label primary amines on proteins in the vicinity of (but not necessarily in direct contact with) the tagged protein of interest. We anticipated that BirA*-tagged VPS33A and VPS33B would result in biotinylation of stable tethering complex subunits and markers of membrane identity.

Wild-type and mutant VPS33A and VPS33B were C-terminally tagged with FLAG-BirA*, and stably integrated into the tetracycline-inducible HEK293 Flp-In T-REx cell line. As expected, when expressed in this cell line, wild-type VPS33A-FLAG-BirA* was able to co-immunoprecipitate endogenous VPS16, while the VPS33A Y438D I441K mutant could not (Fig. 1B). Similarly, wild-type VPS33B-FLAG-BirA* was able to co-immunoprecipitate endogenous VIPAR (Fig. 1B). The VPS33B K419D L431K mutant entirely disrupted binding to endogenously expressed VIPAR, while the VPS33B P428D L431K mutant retained some binding (Fig. 1B). We thus proceeded to use VPS33A Y438D I441K and VPS33B K419D L431K mutants as negative controls for their respective wild-type constructs, thereby facilitating the identification of interactions formed only when VPS33A and VPS33B were correctly assembled into their cognate multi-subunit tethering complexes: CORVET and HOPS for VPS33A, and with VIPAR (as part of CHEVI) for VPS33B.

High-confidence proximity interactions (preys) in BioID assays were considered as having a Bayesian false discovery rate (BFDR) of ≤ 0.01 (i.e., ≤ 1%, as determined by SAINT analysis [24]) and at least twofold enrichment of the protein in the wild-type compared to the negative control mutant. Of these high-confidence preys, very few proteins (20 of 285; 7%) were captured by both VPS33A and VPS33B (Fig. 1C). The majority of preys were found exclusively with either VPS33A or VPS33B, providing strong evidence that VPS33A and VPS33B occupy distinct subcellular locations.

Proteins identified for VPS33A included two of the common subunits of CORVET and HOPS, VPS16 and VPS18, demonstrating that the placement of the BirA* tag did allow for biotinylation of central complex subunits (Fig. 1D). We also identified VPS8, a subunit specifically found in CORVET. Most of the subunits of CORVET and HOPS (excluding VPS33A) follow a similar architecture: an N-terminal β-propeller, an α-solenoid, and (often) a C-terminal zinc-finger domain [2], [25]. Although the mass spectrometry results for VPS33B included VIPAR (gene name VIPAS39), we did not find any other proteins with a domain structure reminiscent of that of CORVET or HOPS subunits (a β-propeller followed by an α-solenoid) as high-confidence preys.

In our BioID results for VPS33A, we identified two out of three of the core subunits of class III phosphatidylinositol 3-kinase (PI3KC3) complexes—PIK3C3 (a.k.a. Vps34) and PIK3R4 (a.k.a. Vps15). PI3KC3 complexes are found on Rab5- [26] and Rab7-positive membranes [27], including early endosomes and late endosomes or autophagosomes (reviewed in Ref. [28]) where CORVET and HOPS function, respectively. We also identified NRBF2, a regulator of the PI3KC3-C1 complex [29], [30], [31]. Furthermore, we identified UVRAG, which is a member of the PI3KC3-C2 complex and is a known interactor of HOPS during autophagosome–lysosome fusion [32]. RUBICON (gene name RUBCN), an inhibitor of PI3KC3-C2 complexes, was also identified as a VPS33A prey. RUBICON reportedly binds directly to UVRAG and prevents UVRAG from binding to the HOPS complex [33], [34]. Our observation that VPS33A can capture RUBICON indicates that UVRAG may not be required for HOPS recruitment to these membranes.

For VPS33B, we observed one previously known interacting protein as a high confidence prey—SEC22B. SEC22B is a SNARE protein with functions in delivering ER-resident proteins to phagosomes in dendritic cells [35] and in plasma membrane expansion [36]. It has recently been described as interacting with VPS33B during the formation of α-granules in megakaryocytes [37].

**Fig. S1:**
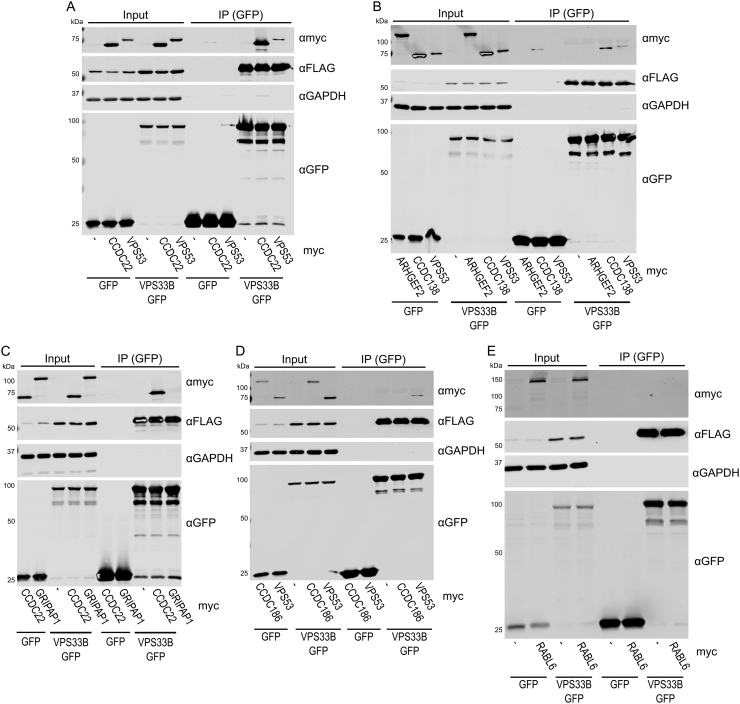
Validation of short-listed candidate VPS33B-interacting proteins. (A–E) ARHGEF2, CCDC22, CCDC138, CCDC186, GRIPAP1, RABL6 and VPS53 (with N-terminal myc tags) were each co-transfected with VPS33B-GFP and FLAG-VIPAR into HEK293T cells. A GFP immunoprecipitation (IP) was performed and samples were analyzed by immunoblotting. Note that RABL6 appears above the expected size (81.3 kDa), as has been observed previously [65]. Furthermore, note that the buffer conditions used for the IP experiment contained EDTA, which may interfere with GDP/GTP binding by RABL6 and thus its ability to bind other proteins.

As the BioID results for VPS33A included many interactors that have already been characterized, we focused on validating our high confidence hits from VPS33B, most of which had not been described previously. Seven proteins were selected for further investigation, based on their high apparent abundance in the mass spectrometry analysis and known involvement in membrane trafficking processes. N-terminal myc-tags were added to the shortlisted proteins ARHGEF2, CCDC22, CCDC138, CCDC186, GRIPAP1, RABL6 and VPS53. These were each co-transfected with VPS33B-GFP and FLAG-VIPAR into human embryonic kidney 293T (HEK293T) cells, and then a GFP immunoprecipitation was performed (Fig. S1). Two of the shortlisted proteins were found to co-immunoprecipitate with VPS33B-GFP under these conditions: myc-CCDC22 and myc-VPS53 (Fig. S1A). Furthermore, over-expression and co-immunoprecipitation of either of these proteins did not affect the co-immunoprecipitation of FLAG-VIPAR, suggesting that this interaction with VPS33B was not competing with VIPAR binding.

CCDC22 is a subunit of the CCC complex (also called the “Commander” complex), along with CCDC93 and COMMD proteins [38], [39]. The CCC complex has been found to interact with other multi-subunit complexes: WASH, Retromer and Retriever [38], [40], [41], [42]. VPS53 is a subunit of both the Golgi-associated retrograde protein (GARP) and endosome-associated recycling protein (EARP) complexes. Both GARP and EARP have four known subunits, sharing three common subunits (VPS51, VPS52 and VPS53) and each containing one unique subunit (VPS54 in GARP, VPS50 in EARP) [43]. Interestingly, VPS51 was also a lower-confidence result in our BioID data set for VPS33B, and VPS52 was a lower-confidence result for VPS33A.

**Fig. 2:**
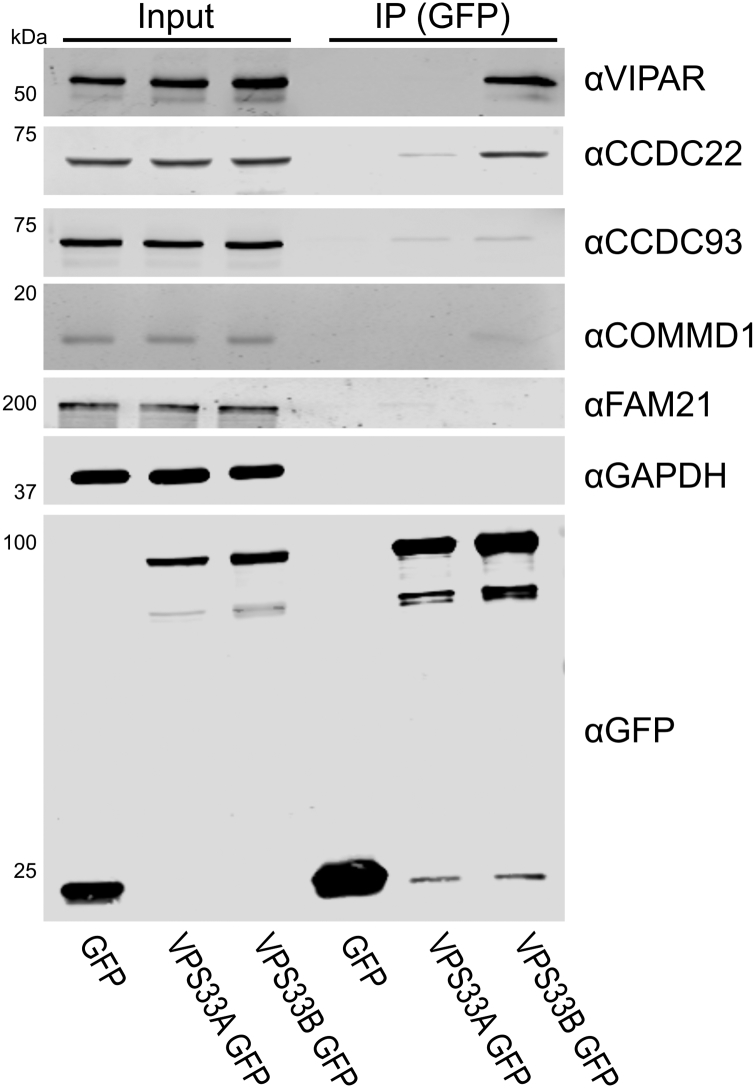
Endogenous CCDC22 is co-immunoprecipitated by VPS33B-GFP. (A) HEK293T cells were transfected with VPS33A-GFP or VPS33B-GFP. After immunoprecipitation (IP), samples were immunoblotted for endogenous proteins using the antibodies indicated.

To probe whether GFP-tagged VPS33B was competent to bind endogenous CCDC22, an immunoprecipitation was performed using only transfected VPS33B-GFP (Fig. 2). Endogenous CCDC22 was efficiently co-immunoprecipitated by VPS33B-GFP. The endogenous CCC complex proteins CCDC93 and COMMD1 were not efficiently captured, nor was the WASH complex protein FAM21. Unfortunately, the lack of a suitable antibody prevented us from testing for co-immunoprecipitation of endogenous VPS53.

**Fig. 3:**
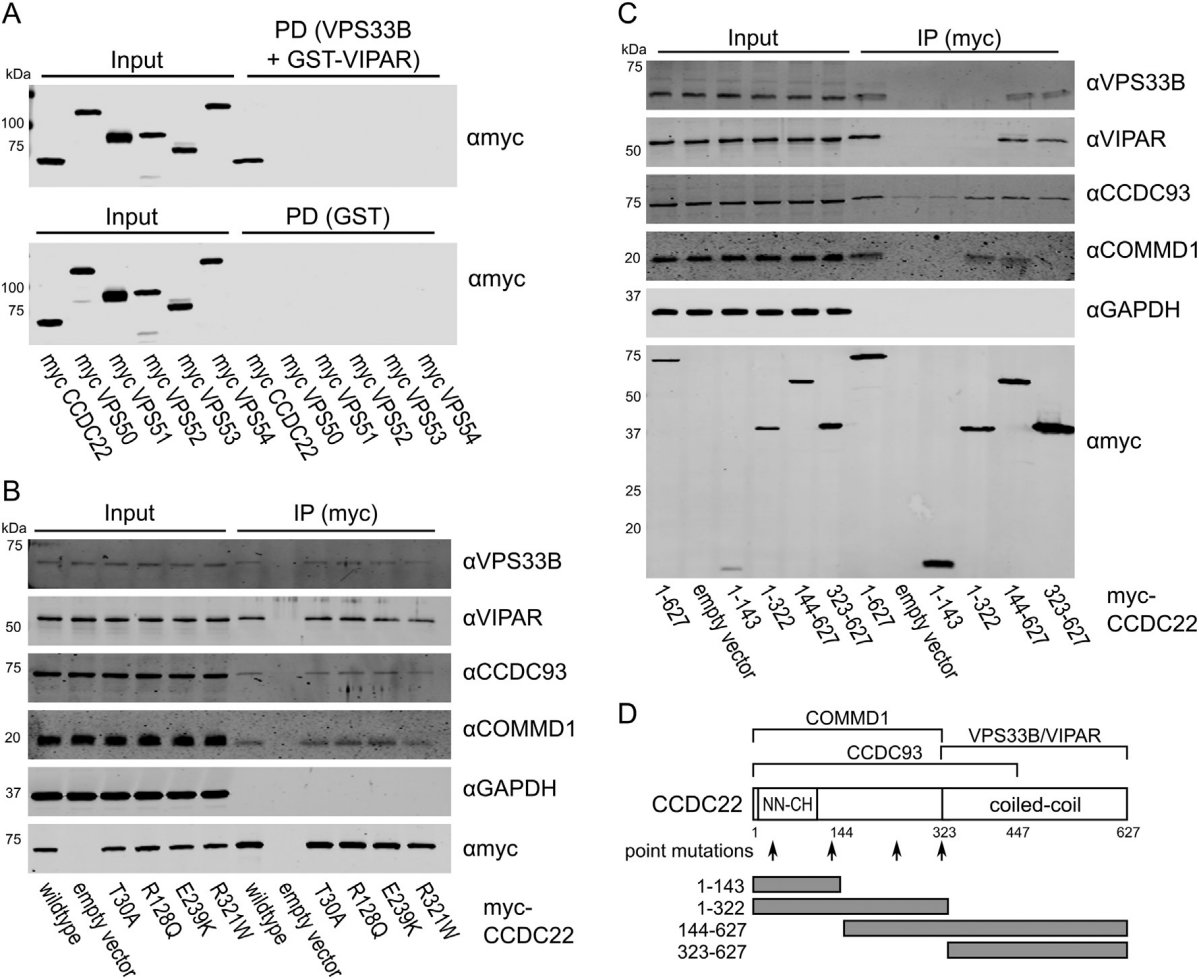
The C-terminal coiled-coil region of CCDC22 binds directly to VPS33B/GST-VIPAR. (A) Myc-tagged CCDC22 or GARP and EARP subunits were produced by *in vitro* transcription/translation and then subjected to GST pull-down (PD) using VPS33B/GST-VIPAR or GST alone. Samples were analyzed by immunoblotting with anti-myc. CCDC22 is efficiently pulled down by VPS33B/GST-VIPAR, but no GARP or EARP subunits are pulled down. (B) Myc-tagged CCDC22 with the wild-type sequence or clinically relevant point mutations [44], [45], or the equivalent amount of empty vector, were transfected into HEK293T cells. After immunoprecipitation (IP), samples were immunoblotted for endogenous proteins using the antibodies indicated. (C) Full length (1–627) and truncated myc-CCDC22, or the equivalent amount of empty vector, were transfected into HEK293T cells. After IP, samples were immunoblotted for endogenous proteins using the antibodies indicated. (D) Schematic of CCDC22, showing predicted N-terminal calponin homology (CH)-like (NN-CH) and C-terminal coiled-coil domains [38], [45], [63], [64], regions required for binding to COMMD1 and CCDC93 [38], [45], the region found to bind VPS33B/VIPAR in this study, and constructs used in this paper.

**Fig. S2:**
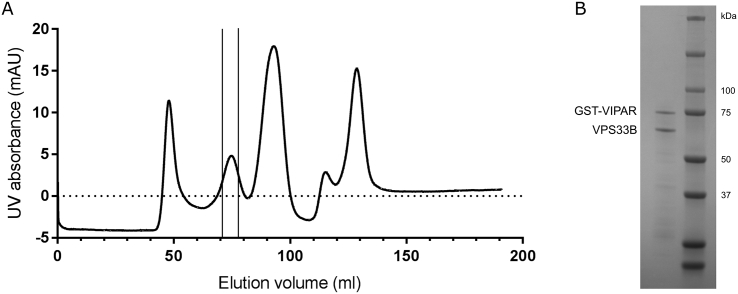
Co-expression and purification of VPS33B and GST-VIPAR. (A) Size exclusion chromatogram (Superdex 200 10/300 GL) showing purification of the VPS33B/GST-VIPAR complex from *E. coli* following GSH affinity purification. Fractions from the indicated peak were pooled. (B) Pooled and concentrated fractions were analyzed by SDS-PAGE. The indicated bands were confirmed by mass fingerprinting as indicated.

In order to perform *in vitro* binding experiments, VPS33B and GST-VIPAR were co-expressed in *Escherichia coli* and purified by GSH affinity and size exclusion chromatography (Fig. S2). The VPS33B/GST-VIPAR complex was used as bait in pull-down experiments, with myc-CCDC22 and myc-VPS53 expressed by cell-free *in vitro* transcription/translation in wheat germ lysate. Myc-CCDC22 was very efficiently pulled down by VPS33B/GST-VIPAR, whereas myc-VPS53 was not pulled down (Fig. 3A). This suggests that either the interaction between VPS53 and the VPS33B–VIPAR complex is indirect (i.e., other proteins contribute to the interaction) or it requires a post-translational modification not conferred in the plant cell-free expression system. To test the former, we repeated the pull-down experiment with each of the other subunits of the GARP and EARP complexes (VPS50, VPS51, VPS52, VPS54), each with an N-terminal myc tag. However, none of these singly expressed subunits interacted with VPS33B/GST-VIPAR (Fig. 3A). We also attempted co-expressing all the subunits of GARP or EARP simultaneously, in the hope of reconstituting the complexes, but this expression strategy was inefficient and did not result in binding to VPS33B/GST-VIPAR (data not shown).

**Fig. S3:**
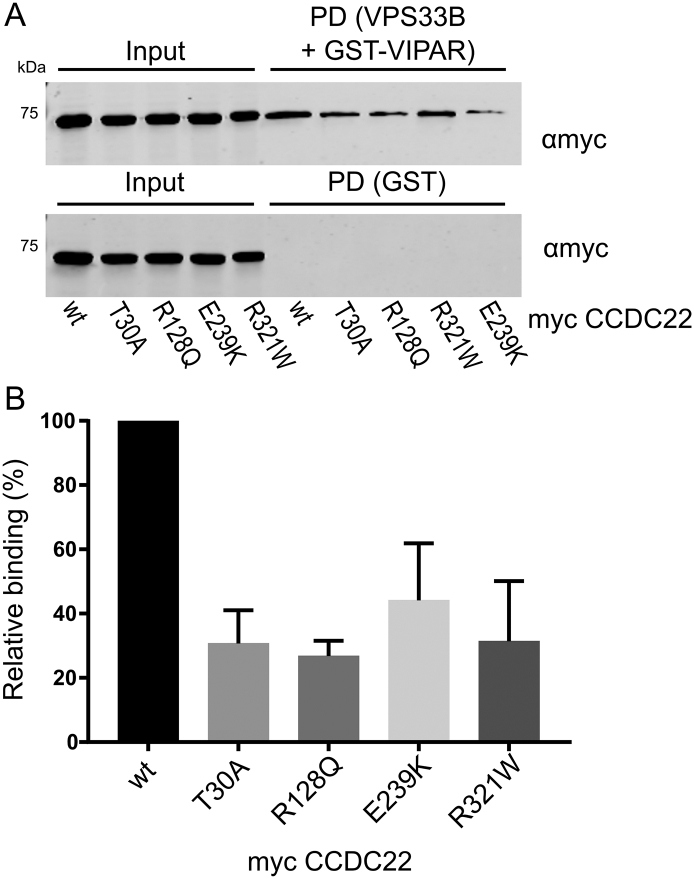
CCDC22 mutants display reduced binding to VPS33B/GST-VIPAR *in vitro*. (A) Myc-tagged CCDC22 was produced by *in vitro* transcription/translation and then subjected to GST pull-down (PD) using VPS33B/GST-VIPAR or GST alone. Samples were analyzed by immunoblotting with anti-myc. Point mutations of CCDC22 demonstrate reduced binding to VPS33B/GST-VIPAR compared to the wild-type (wt) protein. (B) Quantitation of relative binding efficiency across three independent experiments. Immunoblot band intensities were quantified and normalized (intensity of PD band divided by input band, normalized to the PD efficiency of the wild-type construct in each experiment).

Analysis of a cohort of X-linked intellectual disability (XLID) patients identified multiple mutations in the N-terminal half of CCDC22 [44], [45]. One of these mutations (T17A) affects both protein splicing and the interaction of CCDC22 with COMMD1 [44], [45]. None of the other mutations (T30A, R128Q, E239K and R321W) affected binding to COMMD1, but two of these (R128Q and R321W) modified CCDC22 subcellular localization [45]. We therefore wanted to probe whether the correctly spliced mutants bound efficiently to VPS33B/VIPAR. Binding of the CCDC22 point mutants was assayed using the *in vitro* pull-down experiment described above, and each mutant demonstrated reduced binding to VPS33B/GST-VIPAR (Fig. S3). This reduction in binding is consistent either with an interaction between VPS33B/VIPAR and the N-terminal region of CCDC22, or with reduced folding of these mutants *in vitro*. To investigate the latter, wild-type and mutant myc-CCDC22 were expressed in HEK293T cells and co-immunoprecipitation of endogenous VPS33B, VIPAR, CCDC93 and COMMD1 was monitored (Fig. 3B). In this assay, the mutants were all competent to bind both VPS33B/VIPAR and CCC complex components COMMD1 and CCDC93, suggesting that the reduced binding observed *in vitro* arose from impaired folding of the mutant proteins when expressed in isolation.

To further investigate how VPS33B–VIPAR interacts with CCDC22, we generated a series of CCDC22 truncations and probed their ability to bind VPS33B/VIPAR in cells. Full-length and truncated myc-CCDC22 were transfected into HEK293T cells. Subsequent co-immunoprecipitation experiments confirmed that the N-terminal half of the protein mediates its association with members of the endogenous CCC complex (CCDC93 and COMMD1), largely consistent with previous observations although, unlike Phillips–Krawczak and colleagues, we also observed binding of endogenous CCDC93 to the C-terminal region of CCDC22 (323–627) [38], [45]. Co-immunoprecipitation of endogenous VPS33B and VIPAR was observed in the absence of the N-terminal calponin homology-like domain (144–627) or when just the C-terminal coiled-coil domain was present (323–627), but not in the absence of the coiled-coil domain (1–143 or 1–322; Fig. 3C). This suggests that binding to VPS33B/VIPAR is mediated by the CCDC22 C-terminal coiled-coil domain.

**Fig. 4:**
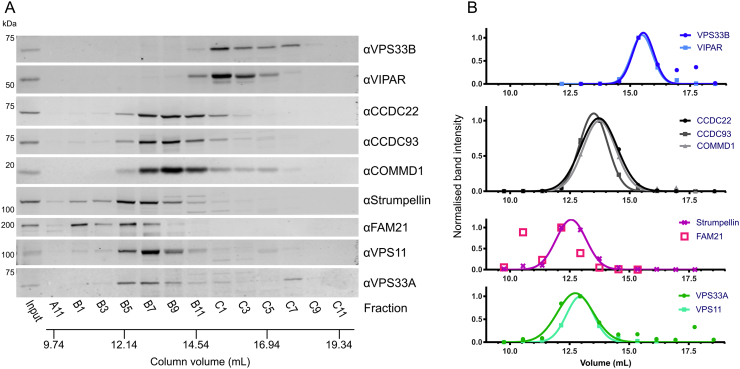
Whole-cell fractionation of HEK293T cells shows that VPS33B and VIPAR form a complex that is considerably smaller than CORVET/HOPS and does not contain CCDC22. (A) HEK293T cell lysates were injected onto a Superose 6 10/300 GL gel filtration column and eluted fractions were analyzed by immunoblotting. (B) Immunoblot band intensities were quantified, normalized to the band of maximum intensity, and fitted to a Gaussian distribution (for all except FAM21, which could not be reliably fitted to a single Gaussian distribution).

**Fig. S4:**
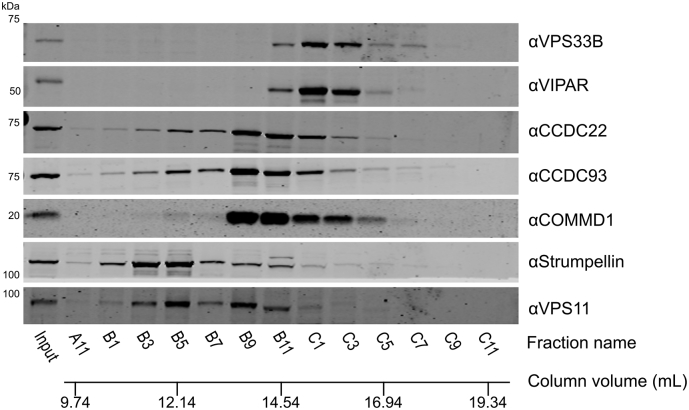
Whole-cell fractionation of HEK293T cells without EDTA is consistent with fractionation conditions containing EDTA. HEK293T cells were prepared with lysis buffer without EDTA. Lysates were injected onto a Superose 6 10/300 GL gel filtration column and eluted fractions were analyzed by immunoblotting using the antibodies indicated.

**Fig. S5:**
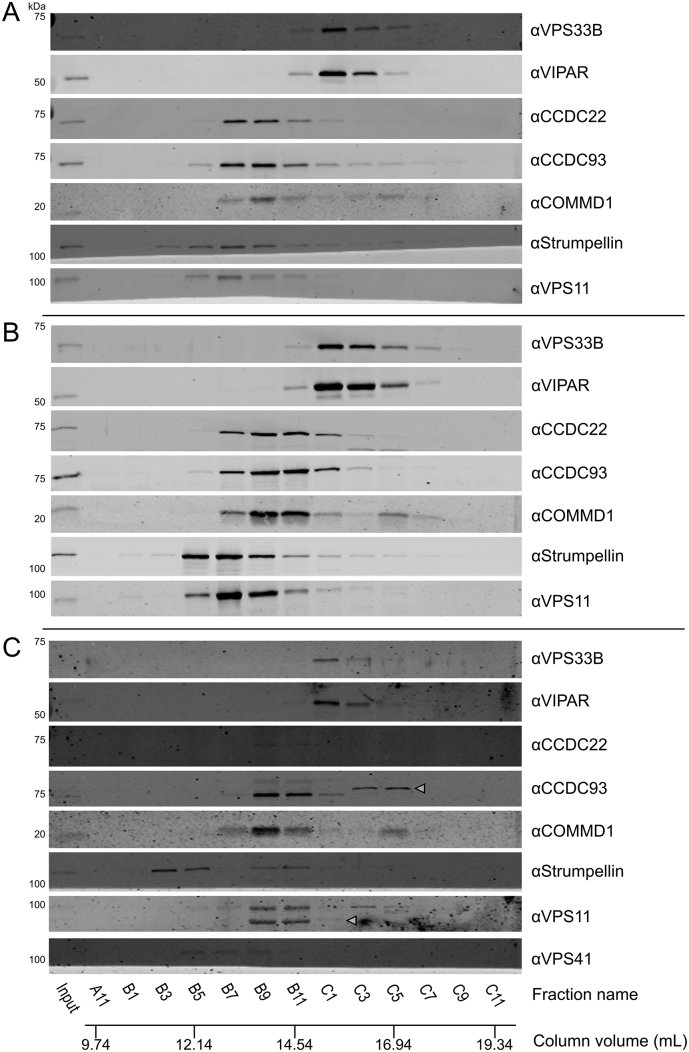
Whole-cell fractionation of HaCaT, THP-1 and HGT-1 cells shows that VPS33B and VIPAR form a complex that is considerably smaller than CORVET/HOPS and does not contain CCDC22. THP-1 (A), HaCaT (B) and HGT-1 (C) cell lysates were injected onto a Superose 6 10/300 GL gel filtration column and eluted fractions were analyzed by immunoblotting using the antibodies indicated. Additional bands at unexpected sizes in panel C, which are presumed to arise due to cross-reactivity of the polyclonal antibodies, are marked with open arrowheads.

In order to determine whether VPS33B and VIPAR form a stable complex with CCDC22 in untransfected cells, we performed a cell fractionation experiment. In brief, whole-cell lysates of HEK293T cells were separated by size exclusion chromatography. A series of elution fractions were collected from the column, the protein content of alternate fractions was concentrated, and the concentrated fractions were analyzed by immunoblotting. In an order from largest to smallest, we observed elution peaks for WASH, CORVET/HOPS, CCC, and then VPS33B–VIPAR complexes during cell lysate fractionation (Fig. 4). It is thus evident that VPS33B and VIPAR do not co-fractionate with the majority of CCDC22 under these conditions. CCDC22 instead elutes in the same fractions as other members of the CCC complex (CCDC93 and COMMD1), proteins that are not efficiently co-immunoprecipitated by over-expressed VPS33B-GFP (Fig. 2). Furthermore, the complex containing VPS33B and VIPAR elutes later than (and is thus considerably smaller than) the CORVET and HOPS complexes, indicating that VPS33B and VIPAR did not elute from the gel filtration column as part of a larger multi-subunit tethering complex. Identical results were obtained when the cell lysis buffer was altered to omit EDTA (Fig. S4), and very similar cell fractionation patterns were obtained using the human cell lines THP-1, HaCaT and HGT-1 (Fig. S5).

## Discussion

In this study, we performed a proteomic comparison of the cellular interaction partners of VPS33A and VPS33B using proximity-dependent biotinylation (BioID). VPS33A was found to co-localize with PI3K3C complex members, confirming previous observations [32], [33], [34]. We also corroborated a recent publication that found VPS33B to co-localize with SEC22B [37]. This confirms that our BioID-tagged constructs were able to localize to the correct intracellular membranes and were competent to label *bona fide* endogenous binding partners. Overall, the majority of proteins identified in our assay co-localized exclusively with either VPS33A or VPS33B, indicating that these proteins are found on different subcellular membranes.

VPS33A is a subunit of both the CORVET and HOPS complexes, the subunits of which share a very similar architecture [2], [25]. While VPS33B-FLAG-BirA* did label VIPAR, we did not find any other proteins proximal to VPS33B that shared the canonical architecture of CORVET and HOPS components, as would have been predicted if the VPS33B/VIPAR-containing CHEVI complex were homologous to these multi-subunit membrane tethering complexes. Furthermore, our whole-cell fractionation experiments performed in multiple human cell lines provide compelling evidence that VPS33B and VIPAR are found in a complex that is considerably smaller than CORVET and HOPS (Fig. 4 and Fig. S5). Given that CORVET/HOPS components co-eluted during these experiments, confirming that such multi-subunit tethering complexes remain assembled under these assay conditions, we conclude that VPS33B and VIPAR do not assemble into a large, stable multi-subunit tethering complex under basal conditions in cultured human cells. We thus recommend that the term “CHEVI” be used to denote the VPS33B–VIPAR bimolecular complex specifically.

VPS33B and VIPAR have previously been described as acting in recycling pathways [13], [14], as has VPS53 as part of GARP and EARP complexes [43], [46], and CCDC22 as part of the CCC complex [38], [40], [41], [42]. Both VPS53 and CCDC22 associated with VPS33B and VIPAR when co-overexpressed (Fig. S1). We were unable to observe a direct interaction between VPS53 and VPS33B/GST-VIPAR *in vitro* (Fig. 3A), and further studies will be required to confirm the nature of this interaction. GFP-tagged VPS33B was able to co-immunoprecipitate endogenous CCDC22 (Fig. 2), and this interaction could be recapitulated using purified recombinant components (Fig. 3A), suggesting that there is a direct physical interaction between VPS33B/VIPAR and CCDC22. Myc-tagged CCDC22 was able to co-immunoprecipitate VPS33B and VIPAR, and truncation mapping showed that the C-terminal coiled-coil region of CCDC22 is required to sustain this interaction (Fig. 3C). Correspondingly, mutations in the N-terminal half of CCDC22 that have been found in XLID patients do not impair myc-CCDC22 binding to VPS33B/VIPAR (Fig. 3B). In agreement with the results presented here, a recent large-scale proteomic study also identified CCDC22 as interacting with VPS33B–VIPAR [47]. We do not observe co-immunoprecipitation of VPS33B-GFP with other members of the CCC complex (Fig. 2). Furthermore, we do not observe efficient co-fractionation of endogenous VPS33B–VIPAR with CCDC22 from cultured human cell lysates, with CCDC22 instead co-fractionating with other CCC complex components (Fig. 4). We note that only stable complexes are likely to co-fractionate in this assay, as (for example) the CCC complex did not co-fractionate with the WASH complex despite these complexes being known to interact [38], [41]. Taken together, our results suggest that the interaction between VPS33B–VIPAR and CCDC22, either alone or as part of the CCC complex, is either weak or transient in cultured cells. We hypothesize that this interaction may be enhanced upon the addition of an external stimulus, for example during bacterial infection, when VPS33B/VIPAR activity has been demonstrated to be important for a robust immunological response [48], [49], [50].

## Experimental Procedures

### Expression constructs/cloning

Human VPS33A and VPS33B were cloned into pDONR223 (Invitrogen) and then pDEST-pcDNA5-BirA-FLAG [51] using Gateway cloning. VPS33A Y438D I441K, VPS33B K419D L431K and VPS33B P428D L431K were created by site-directed mutagenesis of wild-type constructs in pDONR223, then transferred into pDEST-pcDNA5-BirA-FLAG. For transient transfection, wild-type VPS33A and VPS33B were cloned into pEGFP-N1 (Clontech), in order to add a C-terminal green fluorescent protein tag. An N-terminal FLAG-tag was added to VIPAR and cloned into pF5K CMV-neo (Promega). ARHGEF2 (isoform 1), CCDC22, CCDC138 (isoform 1), CCDC186, GRIPAP1 (isoform 1), RABL6 (isoform 1) and VPS53 (isoform 1) were cloned with N-terminal myc tags into pF5K CMV-neo. For co-expression in *E. coli*, DNA encoding VPS33B and VIPAR that had been codon-optimised for bacterial expression (GeneArt) was cloned into positions 1 and 2 (respectively) of the polycistronic vector pOPC [52], VIPAR being tagged at the N terminus with GST. For *in vitro* transcription/translation, full-length human CCDC22, VPS50, VPS51, VPS52, VPS53 and VPS54 were cloned into pF3A WG (BYDV) (Promega) with an N-terminal myc tag. Truncation and point mutations of CCDC22 were created by inverse PCR or site-directed mutagenesis.

### Cell culture and transfection

Untransfected HEK293 Flp-In T-REx cells were grown in Dulbecco's modified Eagle medium with high glucose (Sigma, cat. D6546), supplemented with 10% (v/v) heat-inactivated foetal calf serum, 2 mM l-glutamine, 100 IU/ml penicillin, 100 mg/ml streptomycin, 3 μg/ml blasticidin and 100 μg/ml zeocin, in a humidified 5% CO_2_ atmosphere at 37 °C. Cells were co-transfected with pOG44 (encoding the Flp recombinase) and the VPS33A or VPS33B constructs in pDEST-pcDNA5-BirA-FLAG vectors (described above) using TransIT-LT1 (Mirus), following the manufacturer's instructions. Stable cell lines were selected by addition of 200 μg/ml hygromycin.

HEK293T (ATCC #CRL-3216) and HaCaT [53] cells were grown in Dulbecco's modified Eagle medium with high glucose, supplemented with 10% (v/v) heat-inactivated foetal calf serum, 2 mM l-glutamine, 100 IU/ml penicillin and 100 mg/ml streptomycin. HGT-1 cells [54] were grown in the same medium, supplemented with 10 mM non-essential amino acids (Ala, Asn, Asp, Pro, Ser and Glu). THP-1 cells [55] were grown in RPMI-1640 medium supplemented with 10% (v/v) heat-inactivated foetal calf serum, 2 mM l-glutamine, 100 IU/ml penicillin and 100 mg/ml streptomycin. All cells were cultured in a humidified 5% CO_2_ atmosphere at 37 °C. Cells were transfected with TransIT-LT1 (Mirus), following the manufacturer's instructions.

### BioID and mass spectrometry analysis

BioID and mass spectrometry analyses were performed essentially as described [56]. Briefly, stable HEK293 Flp-In T-REx cells were grown on 15-cm plates to approximately 75% confluency. Bait expression and proximity labeling was then induced simultaneously by addition of tetracycline (1 μg/ml) and biotin (50 μM) for 24 h. Cells were collected in PBS and biotinylated proteins were purified by streptavidin–agarose affinity purification. Proteins were digested on-bead with sequencing-grade trypsin in 50 mM ammonium bicarbonate (pH 8.5). Peptides were then acidified by the addition of formic acid (2% (v/v) final concentration) and dried by vacuum centrifugation. Dried peptides were suspended in 5% (v/v) formic acid and analyzed on a TripleTOF 5600 mass spectrometer (SCIEX) in-line with a nanoflow electrospray ion source and nano-HPLC system. Raw data were searched and analyzed within ProHits LIMS [57] and peptides matched to genes to determine prey spectral counts [58]. High-confidence proximity interactions (BFDR ≤ 0.01) were determined through SAINT analysis [24] implemented within ProHits. Bait samples (biological duplicates) were compared against 12 independent negative control samples (6 BirA-FLAG only and 6 triple-FLAG only expressing cell lines). The specific control samples used in this study were previously published as part of Chapat *et al*. [56]. Dotplots were prepared in ProHits-Viz [59]. Mass spectrometry data have been deposited in the MassIVE database (ID MSV000081814; available for FTP download at ftp://massive.ucsd.edu/MSV000081814) and with the ProteomeXchange Consortium (identifier PXD008457) [60]. A summary of the results is attached as supplemental data.

### Recombinant protein expression and purification

*In vitro* protein expression was performed using TNT SP6 High Yield Wheat Germ reaction mix (Promega) as per the manufacturer's instructions.

VPS33B and GST-VIPAR were co-expressed in *E. coli* B834(DE3). Bacteria were grown in 2 × TY medium to an A600 of 0.8–0.9 at 37 °C and cooled to 22 °C, and protein expression was induced by the addition of 0.2 mM IPTG. After 16–18 h, cells were harvested by centrifugation at 5000*g* for 15 min and the pellet was stored at − 20 °C until required.

Cells were thawed and resuspended in 20 mM Tris (pH 7.5), 300 mM NaCl, 0.5 mM MgCl_2_, 1.4 mM β-mercaptoethanol and 0.05% Tween 20, supplemented with 400 units of bovine DNase I (Sigma-Aldrich) and 200 μl of EDTA-free protease inhibitor mixture (Sigma-Aldrich) per 4–8 L of cell culture. Cells were lysed at 24 kpsi using a TS series cell disruptor (Constant Systems) and lysates were clarified by centrifugation at 40,000*g* for 30 min at 4 °C. Cleared lysate was incubated with glutathione sepharose 4B (GE Healthcare) for 1 h at 4 °C, the beads were washed with 20 mM Tris (pH 7.5), 300 mM NaCl and 1 mM DTT, and bound protein was eluted in wash buffer supplemented with 25 mM reduced glutathione. Protein was injected onto a Superdex 200 10/300 GL column (GE Healthcare) equilibrated in 20 mM Tris (pH 7.5), 200 mM NaCl and 1 mM DTT. Fractions containing purified proteins were concentrated using 30-kDa nominal molecular mass cutoff centrifugal filter units (Millipore), diluted in glycerol to a final concentration of 50% (v/v) glycerol and stored at − 20 °C until required. Purified protein complex identity was verified by peptide mass fingerprinting.

### Co-immunoprecipitations and pull-downs

FLAG immunoprecipitations were performed by harvesting cells and lysing in 10 mM Tris–HCl (pH 7.5), 150 mM NaCl, 0.5 mM EDTA, 0.5% NP-40 and EDTA-free protease inhibitor cocktail (Sigma). Protein concentration in the resulting lysates was quantified by BCA assay (Thermo Scientific), and protein concentration was equalized before immunoprecipitation with anti-FLAG M2 magnetic resin (Sigma). One milliliter of whole-cell lysate, containing approximately 1.9 mg of protein, was incubated with 30 μl of anti-FLAG resin for 1 h at 4 °C. Samples were then washed three times in wash buffer [10 mM Tris–HCl (pH 7.5), 150 mM NaCl, 0.5 mM EDTA, 0.5% NP-40], then eluted by incubation with 250 μg/ml FLAG peptide (Sigma, cat. F3290). GFP and Myc immunoprecipitations were performed using GFP-Trap and Myc-Trap resin (ChromoTek), respectively, as in [61]. GST pull-down experiments were performed as described in [61], using 40–100 pmol purified VPS33B/GST-VIPAR complex as bait (described above).

### Antibodies and immunoblotting

The following primary antibodies were used for immunoblotting: polyclonal anti-GFP (Sigma, cat. G1544), monoclonal anti-FLAG M2 (Sigma, cat. 080 M6035), monoclonal anti-myc (Millipore, cat. 05-724), monoclonal anti-GAPDH (Life Tech, cat. AM4300), polyclonal anti-VPS33A (as described in Ref. [11]), monoclonal anti-VPS41 (Santa Cruz, cat. sc-377118), polyclonal anti-VPS11 (Proteintech, cat. 19140-1-AP), monoclonal anti-VPS33B (Santa Cruz, cat. SC-398322), monoclonal anti-VIPAR (a.k.a. anti-SPE39, gift from S.W. L'Hernault, [62]), polyclonal anti-VPS16 (Santa Cruz, cat. sc-86,939), polyclonal anti-CCDC22 (Proteintech, cat. 16636-1-AP), polyclonal anti-CCDC93 (Proteintech, cat. 20861‐1-AP), polyclonal anti-COMMD1 (gift from E. Burstein, as described in Ref. [38]), polyclonal anti-FAM21 (Santa Cruz) and polyclonal anti-Strumpellin (Santa Cruz). An anti-VPS53 antibody (Sigma cat. HPA024446, lot A106517) was tested but failed our validation assays for immunoblotting. IRDye 800CW-conjugated secondary antibodies were supplied by LI-COR: goat anti-mouse (cat. 925-32210 or 926-68020), goat anti-rabbit (cat. 925-32211), and donkey anti-rabbit (cat. 925-32213 or 926-68023).

All samples were immunoblotted as per [61]. Immunoblot band intensity was quantified using Image Studio Lite (version 5.4, LI-COR) and non-linear fitting to a Gaussian distribution was performed using Prism 7 (GraphPad).

### Whole-cell fractionation

HEK293T, THP-1, HaCaT or HGT-1 cells were harvested and lysed in a buffer similar to that used for immunoprecipitation experiments: 10 mM Tris (pH 7.5), 150 mM NaCl, 0.5 mM EDTA (omitted in the experiment shown in Fig. S4), 0.5% NP-40, EDTA-free protease inhibitor cocktail and Benzonase (Sigma). After centrifugation at 20,000*g* for 10 min, lysates were injected onto a Superose 6 10/300 column (GE Healthcare) equilibrated in 10 mM Tris (pH 7.5), 150 mM NaCl, 0.5 mM EDTA (omitted in the experiment shown in Fig. S4) and 0.5% NP-40. Fractions (0.4 ml) were collected and concentrated using 10 μl of StrataClean resin (Agilent) per 100 μl of fractionated protein. Bound protein was eluted by boiling in SDS-PAGE loading buffer and samples were analyzed by SDS-PAGE and immunoblotting.

The following are the supplementary data related to this article.

Supplementary data Supplementary data
